# Hepatitis C Virus p7 Induces Membrane Permeabilization by Interacting with Phosphatidylserine

**DOI:** 10.3390/ijms21030897

**Published:** 2020-01-30

**Authors:** Hye-Ra Lee, Gi Young Lee, Deok-Gyun You, Hong Kyu Kim, Young Do Yoo

**Affiliations:** 1 Laboratory of Molecular Cell Biology, Graduate School of Medicine, Korea University College of Medicine, Korea University, Seoul 02841, Korea; hyeraleee@gmail.com (H.-R.L.); gylative@gmail.com (G.Y.L.);; 2 Department of Biosystems and Biotechnology, College of Life Sciences and Biotechnology, Korea University, Seoul 02841, Korea; 3 Department of Surgery, Seoul National University College of Medicine, Seoul 03080, Korea

**Keywords:** Hepatitis C virus, p7, membrane permeabilization, phosphatidylserine, gadolinium

## Abstract

Hepatitis C virus (HCV) p7 is known to be a nonselective cation channel for HCV maturation. Because the interaction of HCV proteins with host lipids in the endoplasmic reticulum membrane is crucial for the budding process, the identification of p7–lipid interactions could be important for understanding the HCV life cycle. Here, we report that p7 interacts with phosphatidylserine (PS) to induce membrane permeabilization. The interaction of p7 with PS was not inhibited by Gd^3+^ ions, which have been known to interact with negatively charged lipids, but channel activity and p7-induced mitochondrial depolarization were inhibited by Gd^3+^ ions. From the present results, we suggest that the p7–PS interaction plays an essential role in regulating its ion channel function and could be a potential molecular target for anti-HCV therapy.

## 1. Introduction

Hepatitis C virus (HCV) is a major infectious disease that can cause liver cirrhosis and hepatoma. It comprises a positive-sense single-stranded RNA that encodes three structural proteins (core, E1, and E2) and seven nonstructural proteins (p7, NS2, NS3, NS4A, NS4B, NS5A, and NS5B) [1]. There are seven genotypes with many subtypes, and the most common genotypes are known as genotypes 1 and 2 [2]. For this reason, the 1a H77, 1b BK, 2a J6, and 2a JFH-1 strains have been mainly studied to identify the exact mechanisms of the HCV life cycle [3].

HCV is a good representative example of a virus that interacts with host lipids [4]. In contrast to other viruses that bud from the plasma membrane (e.g., HIV), its life cycle is tightly linked to host lipid metabolism. In terms of its budding process, it has been shown that HCV can interact with lipid rafts composed of saturated lipids with cholesterol [5]. Lipid rafts are predominantly located in the plasma membrane but are also located in the endoplasmic reticulum (ER) membrane [6]. HCV is known to bud from these in an immature form and fuse with lipid droplets (LDs) to form mature particles [7]. For this reason, the relationship between HCV and the ER membrane has been studied in terms of host lipids on the ER membrane. For example, NS5A was shown to interact with phosphatidylinositol 4-kinase alpha (PI4KA), which is important for synthesizing phosphatidylinositol 4-phosphate (PI4P), a precursor of phosphatidylinositol 4,5-bisphosphate (PI(4,5)P2) [8]. PI4KA is recruited on the RNA replication region of the ER membrane, increasing the PI4P level in infected cells [8]. However, direct evidence of an interaction between the HCV-encoded proteins and specific lipids on the ER membrane has been poorly investigated.

It is noteworthy that most viral proteins have multifunctional properties to improve their efficiency in the viral life cycle [9]. For example, HCV p7, a nonstructural membrane protein with two transmembrane domains and an amphipathic alpha-helical transmembrane domain, is known to form a pore [10]. For this reason, its pore-forming activity has been studied with planar bilayer recordings or fluorescent dye-encapsulated large unilamellar vesicles (LUVs). However, its functions in the HCV life cycle are not limited to its ion channel activity. Tedbury et al. suggested that p7 has a function to localize NS2 for assembling virions, and this is not related with its ion channel activity [11]. In addition, it was recently suggested that the novel function of HCV p7 was to play a role in membrane adhesion in the budding process. Lee et al. proposed that HCV p7 can interact with lipid rafts to cause lipid raft adhesion in artificial liposomal systems, and this function was independent of its pore-forming activity [12].

Although HCV p7 has multifunctionality that is a critical factor in the maturation of HCV particles, its lipid preference is not clear. Because the ER membrane is composed of 53% phosphatidylcholine (PC), 20% phosphatidylethanolamine (PE), 11% phosphatidylinositol (PI), 3% phosphatidylserine (PS), 3% sphingomyelin, and 10% cholesterol [13], we hypothesized that HCV p7 might interact with one of the phospholipids that exist in the ER membrane. In this study, we explored the lipid preference of HCV genotype 1a (H77 strain) p7, we suggest that HCV p7 can specifically interact with PS, and this interaction induces membrane permeabilization, which is blocked by nonselective ion channel blockers.

## 2. Results

### 2.1. HCV p7 Specifically Interacts with PS

**Figure 1:**
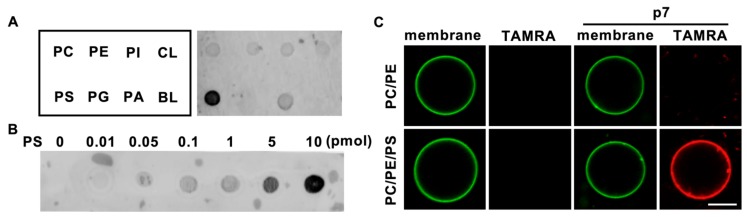
Lipid preference of hepatitis C virus (HCV) p7. (**A**,**B**) Lipid preference of p7 by a protein lipid overlay (PLO) assay. (**C**) p7 interaction with giant unilamellar vesicles (GUVs) composed of PC:PE, 3:1 (mol/mol), or PC:PE:PS, 3:1:1 (mol/mol). GUVs were electroformed with 0.2% TopFluor-cholesterol to visualize the membrane. TAMRA-p7 was applied to examine its targeting to GUVs. The scale bar indicates 10 µm. PC, phosphatidylcholine; PE, phosphatidylethanolamine; PI, phosphatidylinositol; CL, cardiolipin; PS, phosphatidylserine; PG, phosphatidylglycerol; PA, phosphatidic acid; BL, blank.

To investigate the lipid preference of HCV p7, we obtained a 5-carboxytetramethylrhodamine (TAMRA)-labeled p7 synthesized by solid-state peptide synthesis, as reported previously [12], and dissolved it in 50% trifluoroethanol (TFE), which is a solvent for hydrophobic alpha-helical proteins [14]. Natural phospholipids (PC, PE, PI, PS, phosphatidylglycerol (PG), phosphatidic acid (PA), and cardiolipin (CL)) were blotted on the supported nitrocellulose membrane, and a protein lipid overlay (PLO) assay was performed to examine the direct interaction of p7 with phospholipids. Interestingly, we found that p7 specifically interacted with PS (Figure 1A), which is known to have an essential role in the viral life cycle [15], over other cellular phospholipids, and p7 interacted with PS in a concentration-dependent manner (Figure 1B). The PS preference of p7 was examined using giant unilamellar vesicles (GUVs) with or without PS. Consistent with the result of the PLO assay, p7 was targeted to the GUV composed of PC:PE:PS but not targeted to the GUV composed of PC:PE (Figure 1C). This indicates that p7 specifically interacts with PS.

### 2.2. HCV p7 Enhances Membrane Permeabilization in the Presence of PS

**Figure 2:**
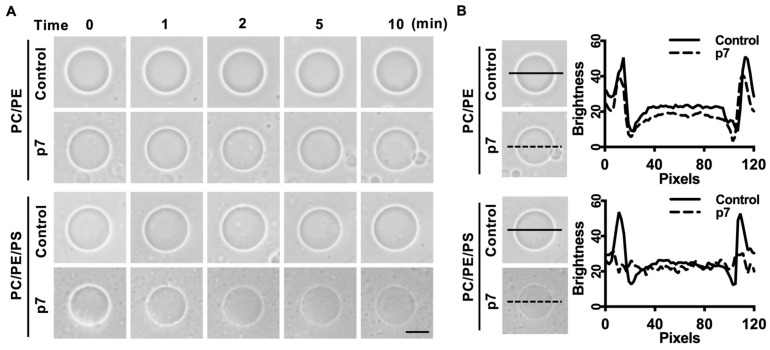
p7-induced giant unilamellar vesicles (GUVs) permeabilization in the presence of PS. (**A**) p7-induced GUV permeabilization. p7 was applied to GUVs composed of PC:PE, 3:1, or PC:PE:PS, 3:1:1, and incubated with external buffer (100 mM KCl, 100 mM sorbitol, 5 mM HEPES/Tris, pH 7). (**B**) Line profiles of p7-treated GUVs. The lines represent cross-sections of the GUVs. Data were analyzed using Zen 2 software. The scales bar indicates 10 µm.

Since p7 has been known to be a pore-forming protein [16], we hypothesized that the interaction between p7 and PS-containing GUV could induce membrane permeabilization. We used phase-contrast microscopy to examine GUV permeabilization. Because GUVs were made with internal buffer (300 mM sucrose) and incubated in external buffer (100 mM KCl, 100 mM sorbitol, and 5 mM HEPES/Tris pH 7), membrane permeabilization could be visualized by the decrease in the phase-contrast effect. As expected, p7 considerably increased the membrane permeabilization of GUV composed of PC:PE:PS when compared with GUV composed of PC:PE (Figure 2A). The decrease in phase contrast was further analyzed by line profiling (Figure 2B).

**Figure 3:**
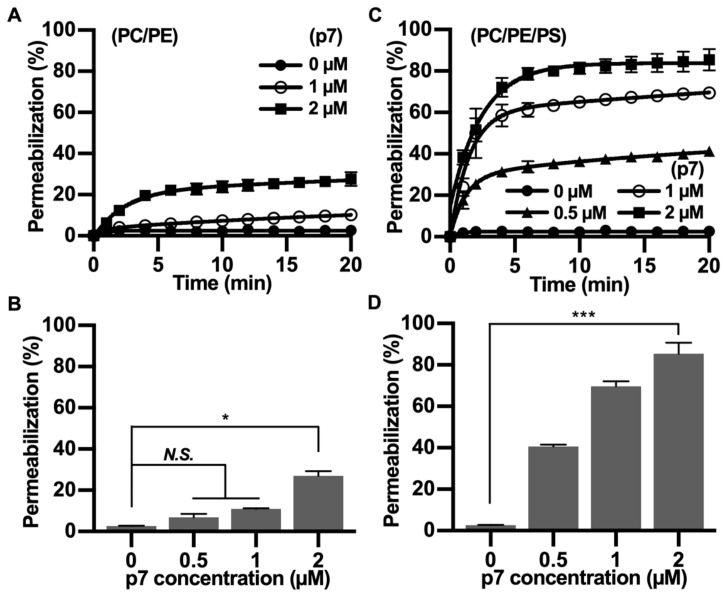
p7-induced large unilamellar vesicles (LUVs) permeabilization in the presence of PS. (**A**,**C**) p7-induced LUV permeabilization. CF-encapsulated LUVs composed of PC:PE, 3:1 (**A**), or PC:PE:PS, 3:1:1 (**C**) were incubated with the indicated concentration of p7, and fluorescent signals were recorded by spectrophotometry (Fluoroskan Ascent FL, Thermo Labsystems, Loughborough, UK). (**B**,**D**) p7-induced liposome permeabilization was quantified at 20 min with CF-encapsulated LUVs composed of PC:PE, 3:1 (**A**), or PC:PE:PS, 3:1:1 (**C**). Data are presented as the mean ± SD and analyzed with 3-way ANOVA. * *p* ≤ 0.05, *** *p* ≤ 0.001. CF, carboxyfluorescein.

Since p7 has been mainly studied with fluorescent dye-encapsulated LUV, we tested the PS preference of p7 with carboxyfluorescein (CF)-encapsulated LUVs composed of PC:PE (Figure 3A,B) or PC:PE:PS (Figure 3C,D). We found that the p7-induced membrane permeabilization was much more potent in PS-containing LUVs, and this result is consistent with Figure 2. Therefore, we suggest that p7-induced membrane permeabilization could be related to an interaction between p7 and PS.

### 2.3. Gd^3+^ Ions Block p7-Induced Membrane Permeabilization

Since p7 has been known to be a nonselective cation channel [17], we examined the effect of Gd^3+^ and La^3+^ ions that are well-known to be nonselective cation channel blockers [18]. To quantify the effects of Gd^3+^ and La^3+^ ions on p7-induced membrane permeabilization, we examined the effects of these with LUVs. N-nonyl-deoxynojirimycin (NN-DNJ), which is known to be a potential HCV p7 blocker [10], was used as a positive control.

**Figure 4:**
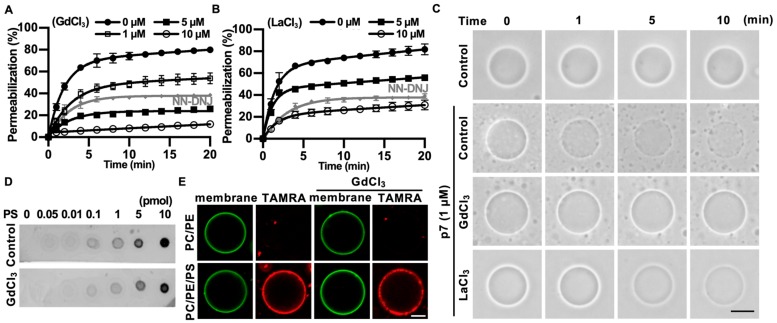
Inhibitory effects of GdCl_3_ or LaCl_3_ on p7-induced membrane permeabilization. (**A**,**B**) CF-encapsulated LUVs composed of PC:PE:PS, 3:1:1 (mol/mol), were incubated with 1 µM p7 and the indicated concentration of GdCl_3_ (**A**) or LaCl_3_ (**B**). Fluorescent signals were recorded by spectrophotometry. NN-DNJ (10 µM) was used as a positive control. (**C**) p7 (1 µM) was used for GUVs composed of PC:PE:PS, 3:1:1, in the presence of 10 µM GdCl_3_ or LaCl_3_ and incubated with external buffer (100 mM KCl, 100 mM sorbitol, 5 mM HEPES/Tris, pH 7). (**D**,**E**) Effects of 10 µM GdCl_3_ on the lipid preference of p7 by PLO assay (**D**) and targeting efficiency to GUV (**E**). The scale bar indicates 10 µm. NN-DNJ, N-nonyl-deoxynojirimycin.

We found that p7-induced LUV permeabilization was clearly blocked by Gd^3+^ ions and was partially blocked by La^3+^ ions (Figure 4A,B). Interestingly, Gd^3+^ ions blocked p7-induced membrane permeabilization more efficiently than 10 µM of NN-DNJ (Figure 4A). These results were confirmed by p7-induced permeabilization with GUVs (Figure 4C). To examine whether these inhibitory effects were caused by an interference in the interaction between p7 and PS, we performed a PLO assay (Figure 4D) and tested the GUV targeting efficiency of p7 (Figure 4E) with or without Gd^3+^ ions. As shown in Figure 4D,E, Gd^3+^ ions had no effect on the interaction between p7 and PS.

### 2.4. Gd^3+^ Ions Block the Channel Activity of p7 in the Bilayer and Inhibit p7-Induced Mitochondrial Depolarization

Next, we examined the effect of Gd^3+^ ions on the channel activity of p7. We used an automated patch-clamp system, “port-a-patch”, which can be used for recording the ion currents from the bilayer directly. To minimize p7 aggregation by salts, we diluted the p7 in 1 M sorbitol (final concentration of p7 = 0.5 µM) and treated the p7 on the preformed bilayer (2-diphytanoyl-sn-glycero-3-phosphatidylcholine(DPhPC):2-diphytanoyl-sn-glycero-3-phosphatidylserine (DPhPS):cholesterol) directly and recorded ion currents of p7.

**Figure 5:**
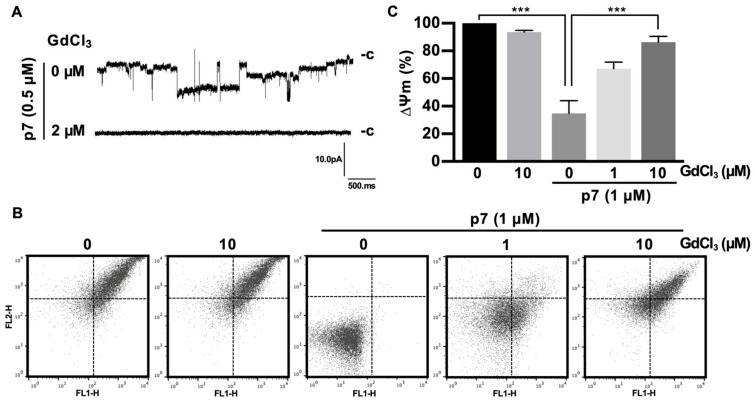
Inhibitory effects of GdCl_3_ on the channel activity of p7 in planar bilayer recordings and on p7-induced mitochondrial depolarization. (**A**) GUVs composed of DphPC:DOPS:cholesterol, 85:5:10 (mol/mol), were electroformed and used to form the bilayer. p7 was diluted with 1 M sorbitol (final concentration of p7 = 0.5 µM) and applied to the preformed bilayer, and then macroscopic currents were recorded. Inhibitory effects of the indicated concentration of GdCl_3_ on the macroscopic currents were recorded. (**B**,**C**) The mitochondria isolated from the mouse liver were incubated with the indicated concentration of GdCl_3_ for 10 min, followed by incubation with 1 µM of p7 for another 10 min, after which mitochondria were stained with JC-1 for 15 min. Mitochondrial membrane potential was recorded with flow cytometry (**B**) and quantified (**C**). Data are presented as the mean ± SD and analyzed with 3-way ANOVA. *** *p* ≤ 0.001.

As expected, Gd^3+^ ions significantly blocked the macroscopic currents triggered by p7 (Figure 5A). Because p7 has been known to target the ER, mitochondria, and plasma membrane, and can cause mitochondrial depolarization in cells [12] and in purified mitochondria [19], we isolated mitochondria from mouse liver and applied Gd^3+^ ions for 10 min, followed by staining with the ∆ψm indicator (JC-1) to examine the inhibitory effect on mitochondrial depolarization. We found that p7 induced mitochondrial depolarization, but Gd^3+^ ions had no significant effect on the membrane potential of intact mitochondria. However, Gd^3+^ ions inhibited the p7-induced mitochondrial depolarization significantly in a concentration-dependent manner (Figure 5B). Figure 5C shows the quantified data of Figure 5B by representing the FL-2/FL-1 ratio of the ∆ψm signal.

Taken together, these results indicate that HCV p7 can interact with PS, resulting in membrane permeabilization, and both the membrane permeabilization and channel activity of p7 can be inhibited by nonselective cation channel blocker Gd^3+^ ions. From the results obtained in this study, we suggest that a p7 inhibitor could be developed to disrupt the p7–PS interaction as a potential molecular target in anti-HCV therapy.

## 3. Discussion

PS is one of the negatively charged phospholipids that account for 5–10% of the host cell membrane [20]. Because of its significance in cellular processes, the identification of interactions between proteins and PS is essential for understanding its regulatory roles [21]. Several PS-binding proteins have been reported [22]; however, only a few studies have reported the interaction between viral proteins and lipids despite its essential function on viral entry and budding [23]. In this study, we showed that HCV p7 prefers PS over other negatively charged phospholipids (PA, PG, PI, and CL). Because PI and PS are major negatively charged lipids in the ER membrane, the interaction between PS and p7 might have essential roles in the HCV life cycle. In contrast to other PS-binding proteins (e.g., annexin V) that need Ca^2+^ ions [24], p7 can interact with PS without Ca^2+^ ions. The interaction between p7 and PS might be explained by its positively charged loop and amphipathic alpha-helical transmembrane domain. It was shown that membrane proteins that have a charged or polar loop can bind to the PS head group by electrostatic interaction, and hydrophobic residues in nearby charged loops can be inserted into membranes [25]. Since HCV p7 has a positively charged loop (K/R-G-K/R) located between two transmembrane domains and is well conserved throughout strains [10], this loop region could interact with the head group of PS. In addition, the nearby hydrophobic residues, such as the amphipathic alpha-helical transmembrane domain, could penetrate the membrane to interact with hydrophobic acyl chains of PS. The alanine substitution of K/R in this loop is thought to impair HCV maturation by affecting the ion channel activity [26], which might be related with the PS preference of HCV p7.

It is noteworthy that p7 can affect the NS2 localization independent of its ion channel activity. This might be related with p7–PS interaction in conjunction with p7–lipid raft interaction. Lee et al. showed that p7 interacted with artificial lipid rafts composed of sphingomyelin: 1-stearoyl-2-oleoyl-sn-glycero-3-phospho-l-serine (SOPS):cholesterol = 65:5:30 (molar ratio) [12]. In addition, Tedbury et al. showed that NS2 was detected in a detergent-resistant membrane fraction such as lipid rafts; however, it was detected in detergent-soluble fraction in the absence of p7 [11]. Thinking that lipid rafts on the ER membrane contain PS, and that NS2 was shown to physically interact with p7, there is a possibility that the p7–PS interaction could lead the NS2 localization in the lipid rafts of the ER membrane to assemble HCV particles. The identification of the PS-binding motif in p7 and the exact function of its interaction with PS in the HCV life cycle needs to be further studied.

Since the main functions of p7 are channel activity and membrane permeabilization, its inhibitors have been screened by bilayer recordings and a membrane permeabilization assay [12]. In this study, we used Gd^3+^ ions to identify its inhibitory effects on p7 function. Gadolinium has been used for magnetic resonance imaging as a form of gadolinium chelate to reduce cytotoxicity caused by free Gd^3+^ ions [18]. Because the ionic radius of free Gd^3+^ ions (107.8 pm) is similar to that of Ca^2+^ ions (114 pm) [27], free Gd^3+^ ions can competitively interact with the Ca^2+^ binding sites of proteins, interfering with vital physiological processes that require Ca^2+^ ions. Free Gd^3+^ ions are also known as inhibitors for nonselective and mechanosensitive channels [28]. However, the inhibitory effects have not been tested for viroporins, viral nonselective ion channels such as HCV p7. We showed that Gd^3+^ ions can inhibit the activity of p7 in all experimental tools we used: LUV/GUV permeabilization (Figure 4A,C), bilayer recordings (Figure 5A), and mitochondrial depolarization (Figure 5B). Its inhibitory effect was much more potent compared to NN-DNJ, which is a well-known p7 inhibitor [29]. Since Gd^3+^ ions can decrease membrane fluidity by binding with negatively charged lipids, Gd^3+^ ions might decrease the channel opening event of p7 by decreasing membrane fluidity. However, Gd^3+^ could not inhibit the interaction between p7 and PS both in the PLO assay and GUV system even though Gd^3+^ ions are known to interact with negatively charged lipids [30]. This might be explained by competitive interactions. The interaction of p7 with PS might be much stronger than that of Gd^3+^ ions with PS. However, the relationship between them needs to be studied further. Although it is not clear that the inhibition of p7 activity by Gd^3+^ ions is related to PS or p7 or both, Gd^3+^ ions could be used for anti-HCV therapy with the controlled release of Gd^3+^ ions from gadolinium chelates to reduce cytotoxicity. It needs to be further studied in HCV culture systems, such as a JFH-1 model, to examine its inhibitory effect on the HCV life cycle.

In conclusion, we have shown that HCV p7 has a preference for PS when targeting the membrane. We suggest that this may be one of the essential factors for p7 function and HCV maturation. A candidate that can inhibit the interaction between p7 and PS, may be a potential candidate for anti-HCV therapy.

## 4. Materials and Methods

### 4.1. Chemicals

Egg PC, egg PE, liver PI, brain PS, egg PG, egg PA, CL, TopFluor-cholesterol (TF-CHOL), DPhPC, DPhPS, 1,2-dioleoyl-sn-glycero-3-phosphatidylcholine (DOPC), 1,2-dioleoyl-sn-glycero-3- phosphatidylserine (DOPS), and 1,2-dioleoyl-sn-glycero-3-phosphatidylethanolamine (DOPE) were purchased from Avanti Polar Lipids (Alabaster, AL, USA). A p7 protein labeled at the N-terminus with TAMRA was chemically synthesized and purified by high-performance liquid chromatography from GL Biochem (Shanghai, China). All other chemicals were purchased from Sigma-Aldrich (St. Louis, MO, USA). The p7 sequence (genotype 1a, H77 strain) is ALENLVILNA^10^ ASLAGTHGLV^20^ SFLVFFCFAW^30^ YLKGRWVPGA^40^ VYAFYGMWPL^50^ LLLLLALPQR^60^ AYA.

### 4.2. PLO Assay

The experiments were performed as previously described with minor modifications [31]. Ten picomoles of PC, PE, PI, PS, PG, PA, or CL were dropped on a supported nitrocellulose membrane (Bio-Rad, Hercules, CA, USA) and dried at room temperature for 3 h. The membrane was blocked with Tris-buffered saline (TBS)-Tween 20 (50 mM Tris-HCl, 150 mM NaCl, and 0.1% Tween 20) containing 0.2% bovine serum albumin (fatty acid free) for 1 h. After washing with TBS three times, the membrane was incubated with 0.5 µM TAMRA-p7 in blocking solution for 2 h. Fluorescent signals were detected on the membrane by a fluorescent image scanner (Typhoon FLA 9500, GE Healthcare Life Sciences, Uppsala, Sweden).

### 4.3. GUV Preparation

PC:PE (10 µL; 3:1, molecular weight) or PC:PE:PS (10 µL; 3:1:1, molecular weight) lipid mixtures with or without 0.5% TF-CHOL were dissolved in chloroform (5 mg/mL) and dried on indium tin oxide-coated glass. The chamber was filled with 300 mM sucrose, and the GUV was prepared using 3 V peak-to-peak and 5 Hz for 2 h at 37 °C using Vesicle Prep Pro (Nanion Technologies GmbH, Munich, Germany). The GUV images were acquired with Zeiss LSM 700 and analyzed using ZEN 2 software (Zeiss GmbH, Jena, Germany). GUV permeabilization was performed in external buffer (100 mM KCl, 100 mM sorbitol, and 5 mM HEPES/Tris, pH 7), and phase-contrast images were acquired with an Olympus CKX31 optical microscope (Olympus, Tokyo, Japan).

### 4.4. LUV Preparation

Ten microliters of the lipid mixture (PC:PE, 3:1, or PC:PE:PS, 3:1:1, mol/mol) was dried in glass bottles and rehydrated with 50 mM CF, 100 mM sucrose, and 5 mM HEPES/KOH (pH 7.4). Multilamellar liposomal suspensions were extruded with a 0.1 µm polycarbonate membrane using an Avanti Mini Extruder and purified by using a PD-10 column (GE Healthcare, Amersham, UK). The p7 with or without GdCl_3_, LaCl_3_, or NN-DNJ was added to LUVs, after which the CF leakage was recorded with a Fluoroskan Ascent FL (Thermo Labsystems, Loughborough, UK) in external buffer (100 mM KCl, 100 mM sorbitol, and 5 mM HEPES/Tris, pH 7). CF leakage was calculated using the following formula:

(1) $$\mathrm{CF}\;\mathrm{leakage}\;(\%)=\frac{F-F_{0}}{F_{\max}-F_{0}}\times 100$$

where F = measured fluorescence intensity, F_0_ = basal LUVs fluorescence intensity, and F_max_ = LUVs treated with 0.1% Triton X-100.

### 4.5. Flow Cytometric Assay

The experiment was approved by the Institutional Animal Care and Use Committee of the Korea University College of Medicine (KOREA-2018-0130, September 26, 2018). Liver mitochondria were isolated from male C57BL/6 mice (6 weeks old) and incubated in buffer containing 250 mM mannitol, 1 mM KH_2_PO_4_, 10 µM ethylene glycol tetra-acetic acid (EGTA), 2 µM rotenone, and 5 mM HEPES/KOH (pH 7.4). The mitochondrial membrane potential was recorded with a FACS Calibur (BD Biosciences, CA, USA). The mitochondria were treated with GdCl_3_ for 10 min, followed by incubation with 1 µM p7 for another 10 min. Mitochondrial membrane potential was recorded after staining with JC-1 for 15 min. The data were analyzed with FlowJo software (Tree Star Inc., Ashland, OR, USA).

### 4.6. Planar Lipid Bilayer Recordings

Bilayer recordings were performed with a Port-a-Patch system (Nanion Technologies GmbH, Munich, Germany) and an EPC-10 amplifier (HEKA Electronik, Lambrecht, Germany). Briefly, electroformed GUVs (DPhPC:DPhPS:cholesterol, 85:5:10, mol/mol) were dropped onto the NPC-1 chip with a pressure of (−) 25 mbars. Symmetric buffers containing 150 mM KCl and 10 mM HEPES/Tris (pH 7) were used for internal and external chambers. After forming a stable seal over 5 Gohm, the chip was overlaid with p7 diluted in 1 M sorbitol, followed by the application of −60 mV. The external buffer was exchanged immediately when the currents were detected. The data were low-pass-filtered at 1 kHz.

## Abbreviations

HCV	Hepatitis C virus
ER	Endoplasmic reticulum
LDs	Lipid droplets
PI4KA	Phosphatidylinositol 4-kinase alpha
PI4P	Phosphatidylinositol 4-phosphate
PI(4,5)P2	Phosphatidylinositol 4,5-bisphosphate
LUVs	Large unilamellar vesicles
PC	Phosphatidylcholine
PE	Phosphatidylethanolamine
PI	Phosphatidylinositol
PS	Phosphatidylserine
PG	Phosphatidylglycerol
PA	Phosphatidic acid
CL	Cardiolipin
TAMRA	5-carboxytetramethylrhodamine
TFE	Trifluoroethanol
PLO	Protein lipid overlay
GUV	Giant unilamellar vesicle
CF	Carboxyfluorescein
NN-DNJ	N-nonyl-deoxynojirimycin
Aman.	Amantadine
DphPC	2-diphytanoyl-sn-glycero-3-phosphatidylcholine
DphPS	2-diphytanoyl-sn-glycero-3-phosphatidylserine
DOPC	1,2-dioleoyl-sn-glycero-3-phosphatidylcholine
DOPE	1,2-dioleoyl-sn-glycero-3-phosphatidylethanolamine
DOPS	1,2-dioleoyl-sn-glycero-3-phosphatidylserine
TF-CHOL	TopFluor-cholesterol
TBS	Tris-buffered saline
EGTA	Ethylene glycol tetra-acetic acid
